# Continuities in State–Society Interactions across Broad Spatio-temporal Realms: Gong Tingxian as Both an Author of Popular Medical Texts and an Imperial Medical Secretary in Early Modern China

**DOI:** 10.1093/shm/hkab036

**Published:** 2022-02-07

**Authors:** Sare Aricanli

**Keywords:** popularisation, China, state–society, continuity, reception

## Abstract

Medical popularisation in late-sixteenth and early-seventeenth-century China has been understood within the context of the state’s retreat from medicine. This article points to ongoing state–societal continuities and thus suggests that the process was more complicated than it had been assumed to be. Analyses with more limited spatial or temporal frames tend to underestimate the extent of continuities forged by societal figures such as Gong Tingxian (fl. 1581–1616), an imperial medical doctor and author of popular medical texts. Gong’s self-fashioning illustrates state–society connections that are also apparent in the reception of his work and in his later legacy. These continuities crossed political, linguistic and cultural lines as Gong’s popular texts were translated to Manchu and housed in a state medical library of the ensuing dynasty. Moreover, Gong’s texts were not only transmitted in China, but reached Japan and Korea, and even found a place within a European collection by the early eighteenth century.

The late sixteenth to early seventeenth centuries in China were a time of flourishing production of popular medical texts by societal actors. Traditionally, this development has been analysed in a context where the state was retreating from medicine. Adopting a different perspective, this article argues that this process was more complicated, and challenges existing understandings of the limited role accorded to the state in medical popularisation.^1^ Examining the reception of texts written by the Chinese medical doctor Gong Tingxian (fl. 1581–1616) across broad temporal and spatial frames allows us to detect the subtle but ongoing continuity of state and society relations.^2^ Such a perspective is crucial, as it tends to be obscured in work that adopts a more regional or chronologically confined point of view. More specifically, this article examines the self-fashioning of the popular medical author’s texts to show how he used his state connections to further his aims of popularisation in society. The analysis that focuses on his later legacy reveals further connections that can be seen through the broad spatial and temporal lens. This study therefore shows that state–society relations were not only top-down, but that societal figures could bring the state back into processes of medical popularisation.

The long-held conviction about the role of the state in early modern Chinese medical popularisation stems from Angela Ki Che Leung’s foundational study on popular medical literature, published in 2003. Leung skilfully showed how popular medical literature in China emerged in the late fourteenth century and that it was promoted by societal actors. The larger context for this, which she discussed in more detail in a 1987 article, was the retreat of the state from medicine and the rise of local actors in southern Chinese society.^3^ This article argues that the connections between medicine in state and society constitute an aspect of early modern Chinese medical popularisation that has been underestimated within the established understanding of local agency in society and a retreating state.

In order to examine these state–society continuities, it becomes necessary to reconsider the relative historiographical emphasis that has been placed on the role of state and society in medical popularisation.^4^ It is also important to bring awareness to assumptions of state medicine as monolithic, and/or an area in which power is only exerted top-down. Recognising the existent multiplicities in state medicine therefore allows for a more flexible structure for evaluating how societal actors made use of their state connections.^5^

The analysis here concentrates mostly on the temporal aspect, with some discussion of the spatial realm. This article examines historical evidence in relation to Gong that extends over dynastic lines, from the Ming (1368–1644) to the Qing (1644–1911) dynasties. Like the more famous eighteenth-century European and American popularisers Tissot and Buchan, Gong’s works also travelled across geographical and political boundaries. While he may not have been as famous as Tissot in the French-speaking realm or Buchan^6^ mostly in the English-speaking world, this article demonstrates that the reception of Gong’s medical works and his historical legacy were not limited to China, and existed across East Asia and in Europe.

Gong Tingxian was a key populariser, and his example constitutes a window onto an early modern Chinese populariser who mediated between state and society. In his study that largely focused on Europe, Roy Porter has drawn attention to critical questions on popularisation, where he has rightly pointed to the importance of understanding culturally specific aspects of this process.^7^ Moreover, he has also addressed the need to examine individual popularisers who acted as go-betweens between the elite and rest of society.^8^

The way authors of popular texts constructed their medical identities provides key evidence of the social spheres to which they claimed to have access. Greenblatt has discussed the processes constituting one’s identity in relation to larger forces acting on individuals.^9^ This study shows that Gong constructed his medical identity by producing popular medical literature which projected himself as being unique while also upholding societal norms and values.^10^ The majority of the examples for Gong’s self-fashioning come from prefaces to his works that are attributed to him and to others. This paratextual evidence, where editorial responsibility is shared by authors and publishers, also prepared the reader for how they should evaluate the particular work at hand.

With its focus on Gong and his popular texts, this study makes contributions to a number of fields. For the history of medicine, it provides an early modern Chinese example of how popularisers mediated between state and society. It builds on earlier scholarship by Leung and Simonis on subjects such as medical organisation and madness in Chinese history that included examples of Gong.^11^ Through an analysis of state–society continuities of medicine from the Ming to the Qing dynasties, it also advances our understanding of early modern China by showing the relevance of Ming dynasty social history for state medicine of the Qing conquest dynasty. Gong’s example, situated in a very particular time and place, also provides one example of the global nature of textual exchange and medicine during early modern times.

This article does not aim to present a comprehensive account of all of works attributed to Gong, his relationship with publishers, numbers of texts printed, economics of printing, or the technologies of print. Neither does it focus on the materiality of the book, aesthetic style, interactions between image and text, or multi-register page layouts. Nor does it aim to document all of Gong’s works in China, East Asia, or Europe.

The study begins by offering background on Gong and his historical context. The analysis of Gong’s medical identity is in the form of a case-study and is structured in two parts. The first presents the construction of his medical identity with attention to connections he claims to state and society. The second part focuses on the reception of Gong’s works and his legacy. The latter section concludes with a discussion of spatial continuities that reflects the regional breadth in the reception of his writings.

## Who Was Gong Tingxian?

Gong was a late-sixteenth, early-seventeenth-century doctor and author of popular medical texts and state medical figure who claimed the identity of an archetypal literati physician. He was a southerner, and a native of Jinxi, Xiasili.^12^ Gong had aimed for an official post. After failing to realise this goal, he turned to medicine. Gong learned through a medical lineage, and expressed a sense of filial piety^13^ in his pursuit of medicine. He made pronouncements of benevolence that are in line with literati ideals,^14^ and self-identified as a literati doctor (more on this further below). Gong also indirectly emphasised the breadth of his medical knowledge by listing a wide range of sources and influences. These included learning from elders, being interested in local medicine, and continuing to study during his travels.^15^ As discussed in more detail below, Gong operated within a context of increasing commercialisation and publishing, where access to knowledge still constituted a challenge. He was inspired by the famous author of medical primers, Li Chan (fl. sixteenth century),^16^ and was one of many doctors and authors who aimed to benefit from the boom in commercial publishing through the genre of popular medical texts. Gong actively participated in the fields of medical practice and publishing. Self-promotion was key to success in both of these areas.

Later in life, Gong held the position of Medical Secretary (*limu*) at the premier state medical institution, the Imperial Medical Bureau (*Taiyiyuan*), which served the emperor’s health, and where his father Gong Xin had held office.^17^ Gong published prolifically through his life, and his texts continued to be reprinted long after his lifetime.^18^

Examining Gong’s main works which are representative of his life and career provides a picture of his trajectory. In 1581, Gong published his own *Effective Potions of Immortality* (*Zhongxing xianfang*). Then, in 1587, Gong produced his major work entitled *Healing Myriad Illnesses* (*Wanbing huichun*). Gong then prepared his father Gong Xin’s *Ancient and Modern Mirror of Medicine* (*Gujin yijian*) (1589–90) for printing. This was followed by *The Divine Archer Forest of Clouds* (*Yunlin shengou*) in 1591. After he successfully treated a member of a princely family, Gong published the *Imperial Prescriptions of the Shandong Prefecture (Lufu jinfang*) in 1594*.* Gong’s first work as a Medical Secretary from 1615 was *Benefiting Society through the Enjoyment of Longevity and Protecting One’s Original [Constitution]* (*Shoushi baoyuan*) (hereafter *Benefiting Society through the Enjoyment of Longevity*). The following year, in 1616, he published the *Complete Book of Benefiting Society* (*Jishi quanshu*). Together, these works reflect the ways in which Gong’s life of publishing popular texts and practicing medicine intersected with Daoist worlds, his father’s training in medicine, his own prowess in treating patients, and his position in the Imperial Medical Bureau.

## The Historical Context of Gong’s Times

The historical context within which Gong published popular texts diverged from that in Europe, as the state did not have much oversight over medicine or publication of popular literature in China.^19^ It was a time of pronounced societal shifts and anxieties, and civil-service examinations constituted the main path to higher status. While the line between state and society is rarely clear-cut, the educational experience through exam-preparation coupled with the consequent failure for many created a literate group of people in society who had more in common with one another through their shared understandings of culture and text.^20^ The exams therefore constituted a central theme in Gong’s prefaces where he addressed his audience.

Medicine as a field had become more scholarly during medieval times, and many of the scholars who failed in official exams then turned to areas such as medicine and publishing. Medicine was a field with no barriers to entry, had a rich textual tradition, and was looked upon somewhat ambiguously but also relatively favourably. It was, therefore, a practical choice for highly-educated gentry, especially toward the end of the Ming. While medical texts had traditionally been those of the classical medical canon, since the late fourteenth century a wealth of popular medical texts had been published. Texts such as Gong’s could be used by those who wished to turn to the study of medicine.^21^

The larger context in which Gong promoted his popular medical texts was that of the flourishing print-culture of early modern China. It was a time of societal flux. While many literati found new careers for themselves, growth in the market was accompanied by shifts in the status of merchants vis-à-vis the literati who were increasingly partaking in the ideals of literati culture.^22^ Rise in status accompanied by increased material production and consumption resulted in social anxieties of declining moral order, attaining new ranks, and maintaining earlier positions. Commercialisation also facilitated the growth of publishing,^23^ and these newly printed texts included those on medicine.

The lack of regulation meant that there were medical practitioners from many different educational and social backgrounds. It was the responsibility of the doctors themselves to define their place in the field. Those with experience of textual learning could claim to be a ‘literati doctor’ (*ruyi*). The term connoted having knowledge of the textual tradition, and was resonant with higher status.^24^ By practicing medicine, those who failed in the examinations could attain a level of social status that was almost, but ‘not quite’ that of a Confucian gentleman.^25^ The now famous quote by Fan Zhongyan (989–1052) ‘If one cannot be a fine minister, one should be a fine doctor’^26^ exemplifies the place medicine held in society through Gong’s lifetime.

In *Healing Myriad Illnesses* Gong emphasised the close parallels between the fields of bureaucracy and medical practice.^27^ Gong wrote that his ambition was to be like the virtuous ministers of state in ancient times, and aid in harmonising the transforming influence of the ruler’s virtue. He continued, in line with Fan Zhongyan’s words, that if you think you cannot become a virtuous minister and support the long life of the nation, ‘then become a virtuous doctor and diagnose peoples’ hardships’ ^28^ to aid in long life of the common people. Gong then continued to praise medicine saying ‘the path of medicine is grand’ and commented on the vast textual corpus.^29^

Gong lived in a society that venerated the textual tradition. He astutely promoted his works within the context of a commercialised publishing sector. He did so by advertising the breadth of his medical knowledge as well as his understanding of the classical medical tradition. He claimed that his works brought together medical knowledge from various social spheres (family medical lineage, master–disciple relationships, medicine of localities, secret medical knowledge, official medical institutions) to which he had access. Such pronouncements were significant as these were areas in which medical knowledge was formulated and transmitted. His ability to obtain a wealth of medical knowledge and his willingness to distribute it carried weight, because even within the context of highly commercialised print-culture, the ability to acquire textual materials still constituted a real challenge.^30^ Private collections were open to a coveted few, and state texts were inaccessible to the general public.^31^ Medical texts were after all very profitable and constituted a sizable proportion of published works.^32^ Gong clearly knew how to turn a challenging situation into one that worked in his favour.

Gong aimed to reach a wide readership, and his audience included a number of groups in addition to aspiring doctors who may have not been able to afford an apprenticeship. His texts were marketed for learning medicine, for self-treatment, or for a doctor to use when he called on patients at home. Gong clearly did not limit his readers to the literati.^33^ The literati would not have needed to read his texts. As Gong’s audience included those rising in society, Gong’s cultural capital, even if self-advertised, was an asset. While Gong often wrote of his desire to make his works accessible to people who were in the lower ranks of society, he was speaking of them rather than to them.^34^

The motivations for dissemination were the values of a gentleman steeped in knowledge of Confucian classics, such as benevolence and helping those in society.^35^ Advertising such traits was one of the strategies he used to reach wider audiences, which would translate into economic gains. While Gong did refer to competition, he hardly broached the matter of finances, which was at the very heart of commercial printing. After all, doing so would not have appeared to be reflective of the very scholarly ideals that he claimed to embody.

The structure, simplified language and content, and practical nature of introductory medical texts facilitated their use and contributed to their popularity.^36^ Rather than theoretical discussions of *qi* and *yin-yang*, or complicated textual commentaries, Gong’s works had pharmaceutical formulas for treatment, and songs and poems to facilitate memorisation of *materia medica*. Providing medical information in an easily memorisable format contributed to the popularity of his works.^37^*Healing Myriad Illnesses*, for example, contained verses in song form *(ge*) on subjects such as *materia medica*, meridians, organs and viscera. It also had sections on paediatrics, and women’s medicine (*furen ke*) which included gynaecology and obstetrics.^38^ Gong increased the appeal of his text *Imperial Prescriptions of the Shandong Prefecture* by appending a short treatise on extending one’s life by 20 years.^39^

The practical nature of introductory medical texts coexisted with their claim to provide a window onto classical medical texts (discussed further below). The language of medical texts was more literary than spoken.^40^ Chen Xiuyuan (1753–1823), in the section of his 1804 text on ‘origins and currents of medical studies’, stated that Gong’s *Healing Myriad Illnesses*, along with other works now considered to be fundamental medical texts were merely shortcuts.^41^ Chen was not alone. In 1831, Wu Tang had complained that people were reading Gong’s *Benefiting Society through the Enjoyment of Longevity* rather than the medical classics.^42^ Chen and Wu’s comments attest to the fact that Gong’s texts continued to gain popularity long after his time.

## Construction of Medical Identity through Gong’s Texts

In considering what kind of a medical identity Gong aimed to construct, it is important to remember that ways to imagine one’s identity abounded at a time of societal flux.^43^ The passages below show that Gong mentioned his family’s ties to state medicine, as well as his later position. Moreover, he secured prefaces from famous authors and officials, and promoted his ties to a Ming princely household. The examples also point to societal, as well as state actors who participated in the process of medical popularisation.

Gong explained that he benefited from others’ medical experience and knowledge. He associated with various respectable experts, and met with knowledgeable elders of the ‘sagely way’.^44^ He stated that he learned medical knowledge from his family,^45^ expressed his earlier desire to succeed in the civil service exams before turning to medicine, and showcased his knowledge of classical medical texts. Gong also emphasised his practical experience.^46^ Gong listed the places of travel where he claimed to have collected medical knowledge,^47^ but did not demonstrate their particular significance to his work, either through organisation of the material or with direct references. As it was common to do, he did mention location in relation to his hometown, Jinxi in Shandong.^48^

In *Effective Potions of Immortality*, Gong stated that medical knowledge passed down in the family could be found in the *Ancient and Modern Mirror of Medicine*.^49^ This text was his father Gong Xin’s as yet unpublished work. In a society that accorded great value to descent, publishing his father’s work after having published his own, was an act of filial piety that further consolidated his father’s legacy, and reaffirmed his family medical lineage.

Securing laudatory prefaces from famous figures was another way Gong constructed his medical identity.^50^ Medical patronage was not only from the very top, but also included princes who provided support for publication.^51^ The Prince of Shandong’s preface and financial contribution for Gong’s *Imperial Prescriptions of the Shandong Prefecture* illustrates this point. According to the text, all the best doctors at court had been called to treat the lady of a princely household in Shandong. While none had been successful, Gong had cured her. The prince gave Gong one thousand pieces of gold which Gong did not accept, an act which he publicised to attest to his high moral ground. The princely household then published Gong’s *Imperial Prescriptions of the Shandong Prefecture*. Gong was designated the ‘best doctor’, and presented with a large horizontal board (*bian’e*) with the inscription ‘number one doctor’, *yilin zhuangyuan*^52^ where *zhuangyuan* referred to the title conferred to the person who came in first in the highest imperial examination. Here, it depicted a leading medical figure whose status was comparable to scholars.

Another famous figure who wrote a preface for Gong was the literary writer and book collector Maokun (1512–1601), who had held posts at the Ministry of Rites and the Ministry of Personnel. One of Maokun’s prefaces was for Gong’s *The Divine Archer Forest of Clouds*.^53^ In describing a doctor’s practice, Maokun used the common metaphor of an archer shooting a bow and hitting the target. The preface stated that only Zhu Danxi and Zhang Zhongjing had preserved and explained the knowledge of Qibo, Huangdi, Cang Gong, and Bian Que. It added that the work sustained itself throughout generations, and was in the tradition of Zhu Danxi and Zhang Zhongjing.^54^ Maokun thus placed Gong within a longer tradition of esteemed doctors from ancient and medieval times.^55^ In the preface, Maokun also promoted Gong’s other works, as Gong himself often did. Moreover, Maokun listed texts Gong had published earlier, and stated that *The Divine Archer* was admired with fear by those near and far, copied out, and that its price reached that of paper in Luoyang (see below), to allude to the printing of many copies.^56^ Maokun praised the book by saying that it included pulses, pediatrics, and secret^57^ pharmaceutical recipes in rhyme form, which facilitated reading out loud.

An alternative strategy that Gong used to construct his medical identity was to showcase his training in classical texts.^58^ By doing so, Gong could aim to attract the readership of those aspiring to literati culture.^59^ For example, when he distinguished between himself and other doctors, he complained about those medical practitioners who lined up around the town, and referred to them with a phrase that means to mistake fish eyes for jade, or to confuse two things that may seem alike but which are clearly very different. According to Gong, such doctors did not miss their mark in getting patients.^60^ Furthermore, in *Complete Book of Benefiting Society* he stated that his earlier work entitled *Benefiting Society through the Enjoyment of Longevity* had cost at least two or three pieces of gold, and many had purchased it, resulting in the ‘paper in both capitals becoming expensive’.^61^ Here he made a reference to ‘paper is dear in [the city of] Luoyang’ (*Luoyang zhi gui*). The saying went back to the Western Jin dynasty (3rd–4th c.), and was used to refer to the wide circulation of a text. It referred Zuo Si’s *Three Capitals Rhapsody* (*Sandu fu*) that was copied so many times as to cause a paper shortage in Luoyang.^62^ Gong changed the phrase from ‘paper in Luoyang’ to one that stated ‘paper in both capitals’ became dear. ‘Both capitals’ referred to Beijing and Nanjing in the Ming dynasty, and so shifted the temporal frame of the reference to his time.

Gong’s words did not always remain limited to the scholarly realm, but also included broader resonances, such as Daoism.^63^ This was partly due to the fact that the learning of his audience was very varied. There was also a more practical reason: the proximity of Gong’s hometown, Jinxi, to the famous Daoist pilgrimage site Mount Longhu.^64^ In *Effective Potions of Immortality* Gong exclaimed that when the impoverished who live in remote locations cannot obtain his work, that is truly like ‘the apricot forest losing the gift of spring’.^65^ By using this phrase, Gong demonstrated his familiarity with the myth in the *Traditions of Divine Transcendents* (*Shenxian zhuan*) from the 3rd to 4th centuries. According to the legend, Dong Feng was an enlightened man, who practiced the Daoist Way. A magistrate who returned to the area after many years noticed his unique ability, as Dong had not aged. Dong also exhibited great skill in medicine. He did not accept payment for treatment, but asked that cured patients plant apricot kernels. The area became covered with a rich forest of apricot trees, thus attesting to his success.^66^ Moreover, the title of the text *Effective Potions of Immortality*, *Zhongxing xianfang*, also reflects Gong’s interest in exhibiting his familiarity with Daoism. The term ‘*zhongxing*’ means to grow apricots (referring to the legend of Dong Feng), and ‘*xianfang*’ is an elixir of immortality, a clear reference to these practices. The title of another one of his works, *The Divine Archer Forest of Clouds,* also invoked such a connection. Forest of Clouds or Yunlin was an abbreviated version of Gong’s literary name The Forest of Clouds Hermit (Yunlin shanren).^67^ The designation hermit or recluse (shanren) in Gong’s literary name, as well as the reference to the cloud motif are suggestive of Daoism. As texts could be published under different titles, and the title page of a late-sixteenth to mid-seventeenth-century edition of *The Divine Archer Forest of Clouds* provides another a good illustration of Gong’s ability to bring together different worlds. It reads, *Imperial Medical Bureau Divine Archer’s Pills of Immortality*, thereby reflecting the convergence of Gong Tingxian an Imperial Medical Bureau doctor with the Daoist world of longevity techniques.^68^

Gong promoted himself as occupying a unique position to access a wide range of medical understandings, and emphasised this by stating that his works contained ‘secret knowledge’.^69^ His *Effective Potions of Immortality* even included an appendix of secret prescriptions—clearly a secret no longer.^70^

Gong’s gesture toward Daoist medical ideas, secret medical knowledge, techniques for longevity, and moxibustion did not represent the breadth of the content in his texts. In addition to the more conventional methods of decoctions, pellets, powders, ointments and fermented medicinal preparations, his work included external therapies. It also featured discussions on dermatological treatments, ‘cosmological numerology’ (*hetu luoshu*) and magical medicine such as ‘evil spirits’ (*xiecheng*).^71^ Gong’s skill was to bring together a breadth of medical knowledge by emphasising his ability to acquire it. While he did not say so, through his contacts in society and state medicine, he not only could obtain the knowledge but also had access to the resources to disseminate it. He expressed his desire to share this knowledge by stating that he was well-educated as a child, and that he humbly received the medical texts passed down in the family and prepared the *Ancient and Modern Mirror of Medicine* for those in society.^72^ He also hoped that *Effective Potions of Immortality* could reach distant corners, and be understood by the people there, allowing everyone to be under the shade of apricot trees, that is, the protection of good doctors.^73^ His desire to disseminate was also visible in *Healing Myriad Illnesses*, where he said that he prepared it for publication as he could not bear to keep the secrets any longer.^74^ Gong certainly valued disseminating his works. One of the factors that supported Gong’s self-promotion was his connection to state medicine.

##  Institutional Context

Gong attained a place in the Imperial Medical Bureau later in life. The usual ways of attaining a position at the Imperial Medical Bureau included taking the civil-service examinations, getting a position through a family member, or being selected from the provinces.^75^ Although it is understandable that Gong failed the provincial civil-service examinations—most people did—one might have thought that he may have attained the position through his father who had been an Imperial Physician (*yuyi*)^76^ while Gong had only reached the level of a Medical Secretary. One explanation for his appointment is that he had been recommended for the post by a certain high official.^77^ Gong’s work in different localities, and his ability to treat a disease that the Imperial Medical Bureau doctors had not been able to cure, had made him an asset.^78^

In order to understand the place that Gong Tingxian held, and his position with respect to his father, it is necessary to turn to the structure of the institution.^79^ Gong was a Medical Secretary, and therefore was not among the highest-ranked officials, such as the Commissioner or two Administrative Assistants. Nor was he among the around 70 members at the lower end of the hierarchy. He was one of the 20 Imperial Physicians and Medical Secretaries. As a Medical Secretary, he could have advanced to the position of Imperial Physician. Imperial Physicians were among the top three ranks that entered the imperial compound. However, he did not attain the title of Imperial Physician that his father had. As a Medical Secretary, Gong was in the middle of the hierarchy in the Imperial Medical Bureau.

## Medical Secretary’s *Benefiting Society through the Enjoyment of Longevity*

One of the main reasons *Benefiting Society through the Enjoyment of Longevity* holds an important place in the construction of Gong’s medical identity is because it is the first book in which he signed his preface as Medical Secretary of the Imperial Medical Bureau. He began this text by praising his childhood education and family background, and explained that after he read the medical work his father wrote, he continued with his studies. He stated that five of his own works were circulating in society.^80^ Being tired of wandering, he closed himself up at home, contemplated the deeper meaning of medicine, and understood more each day. He collected pharmaceutical recipes from different localities, the famous medical texts of the palace, medical books passed down in important families, and secret medical texts kept in homes of non-medical elites. Gong also asserted that he read and collated these texts, adding materials that earlier scholars had not discovered. He took the hundreds of effective medicines and wondrous prescriptions, categorised them, and called the final result *Benefiting Society through the Enjoyment of Longevity*.^81^ Gong asserted that it represented the entirety of the ways of medicine, filling in what was missing in previous treatises. He concluded by saying that he had included discussions on ‘nourishing life’ (*yangsheng*), otherwise how could it be compared to other important medical works?^82^

A characteristic aspect of commercial imprints was the extent of paratextual front matter, which included many prefaces as well as general regulations (*fanli*), namely a foreword.^83^*Benefiting Society through the Enjoyment of Longevity* had both of these paratexts. The general regulations stated that the work ‘had been compiled by honoring the *Yellow Emperor’s Classic of Internal Medicine* [*Huangdi Neijing*], by taking [the ideas of] Liu, Zhang, Zhu [and] Li’ as principal. Moreover, the text added that Gong had ‘*deleted what was complicated, collected what was uncomplicated*, followed the wonderful and virtuous [aspects, and] gathered their meaning’. He continued to explain that he brought the knowledge together to compile this comprehensive volume.^84^ Gong’s general regulations are therefore emblematic of the tension between on the one hand emphasis on the ancient and medieval medical knowledge based on the textual tradition, and on the other hand the simplified nature of popular texts. In this regard, Gong argued that knowledge from classical medical texts was the foundation of his understanding. As mentioned above, in the early nineteenth century, Chen Xiuyuan and Wu Tang criticised Gong for having written works that were shortcuts to reading medical classics.

*Benefiting Society through the Enjoyment of Longevity* begins with a section that aims to set out the principles of medicine, and as such it is very similar to the kind of knowledge presented in a contemporary text on the fundamentals of Chinese medicine. This included discussion of the Chinese organ systems, their excess and deficiency, and topics such as meridians, pulses, Blood and *qi*, as well as references to the medical classics. Some sections are in the form of songs (*ge*) that facilitate memorisation. The text also had information on a wide range of therapies. These included topics such as nourishing life, paediatrics and women’s medicine, dietary therapy and moxibustion.^85^ In an edition where the text *Benefiting Society through the Enjoyment of Longevity* is over 350 pages long, the discussion of medical classics constitutes just one page, and the introduction of fundamentals 20 pages. The section on classics and fundamentals of medicine and a short section on *materia medica* constitute the first chapter. And the remaining nine chapters or over 300 pages cover various treatment methods, clearly showing the focus on practical medical solutions. The pharmaceutical formulas for daily use, and range of topics for treatment (including common illnesses, care of elders, children etc.) made the book a handy manual of medicine. 

Gong’s experience in treating patients was also an integral part of his medical identity. Not all authors of popular medical texts were doctors themselves, but in *Healing Myriad Illnesses* Gong explained that his experience had increased over time, that he treated a range of disorders, and that serious ailments had suddenly healed the way plants and trees grow upon the coming of spring. Gong explained that he had been intellectually nourished by the magnificence and splendour of texts from the early medical sages, examined these in detail, and considered them to formulate his own understanding, which he collected into this book.^86^

Gong’s strategies reflected ways to broaden his readership. He promoted his access to knowledge in state and society, utilised prefaces from famous figures, and showcased his familiarity with classical literacy and Daoist understandings. He also capitalised on the anxieties of those in his audience who had failed to acquire a bureaucratic post, and participated in the broader trend to suggest that they could turn to medicine. Within the context of a flourishing print-culture, difficulty in accessing certain texts, as well as a reverence for textual traditions, he provided useful medical information in the genre of a medical primer.

## Reception and Continuity

Gong’s popular texts enjoyed continued attention through his claims to have access to knowledge within the state and society, and his presentation of a wide range of practical information in easily accessible forms. As a testament to his continued popularity over time, his authorship was attributed to books long after his passing. In 1691, a book titled *Infants’ Complete Book of Secret Decrees for Pediatric Massage and Medicine* (*Xiao’er tuina fangmai huoying mizhi quanshu*), and attributed to Gong was published. The last part of the preface for this work is similar to ways Gong expressed himself in earlier prefaces. In the preface, for example, the author remarked on the challenges associated with treating children with herbal medicine, as their bodies have not yet fully formed, their Blood and *qi* have not stabilised, and their organs and viscera are still weak. The author continued that when children are ill (i.e. have vomiting, diarrhea, cough etc.), only medical massage could permeate the path of Blood by pressing on the meridians of the child’s Five Organs and Six Viscera. He explained that it had been a long time since he had received the secret texts on this method, and that he had had many opportunities to put it into practice. Expressing his desire to no longer keep it to himself, he shared the text with those in society.^87^

While there are some similarities, the authorship raises a number of interesting questions. The date of this preface, 1691, is well beyond Gong’s lifetime. Although there is the possibility that it may have circulated in manuscript form for some time before being published, there is no other evidence from Gong’s lifetime that he has such a text. The language, furthermore, is much simpler than that in Gong’s other works. The text is clearly one attributed to him rather than one written by Gong. Having a text published in his name more than half a century after he likely passed away shows the continuing importance of his legacy into the ensuing Qing dynasty as his name maintained some market value.

While Gong’s work in constructing his medical identity and publishing texts certainly helped him attain fame as can be seen through this text that was posthumously attributed to him, and which circulated in society, there were also some limitations to his strategy. Gong seems much less visible in some of the prominent state compilations. The official *Ming History* (*Mingshi*), for example, did not mention Gong, though it included other authors of medical texts.^88^ Gong could have been part of the *Ming History*, as he is in the bibliography of Wan Sitong’s *Draft Manuscript of the Ming History* (*Mingshi gao*), which lists both his *Healing Myriad Illnesses* and *Effective Potions of Immortality*.^89^ These two texts are also in Huang Yuji’s (1629–1691) famous late-seventeenth-century *Catalogue of the Hall of One Thousand Acres* (*Qianqingtang shumu*),^90^ which formed the basis of the bibliography in both the *Draft Manuscript* and the *Ming History*. In Huang Yuji’s catalogue, right below the titles of Gong’s texts is an annotation. These are his name, hometown, and his official position at the Imperial Medical Bureau, attesting to how the importance of his state connections also solidified the reception of his works. When Wan Sitong’s *Draft Manuscript of the Ming History* was revised into Zhang Tingyu’s final official *Ming History*, however, Gong was no longer in the final version. The revision process took many years, and involved a wide range of people who worked within a politically charged context. Historians have agreed that there are many differences between the two texts.^91^ The fact that Gong was edited out of the text is not so surprising when one considers the process of revision.

Another bibliography that mentioned Gong was that by Zhou Zhongfu (1768–1831). It listed *Healing Myriad Illnesses*, noted that the author was a Medical Secretary at the Imperial Medical Bureau, and recorded that he had cured the lady of the Shandong princely household.^92^ State-ties, therefore, continued to constitute an important aspect of a popular text’s reception.

It was not just Ming official sources or works published in society through the early modern period that included Gong. In fact, some Qing imperial compilations also included him. For example, the *Synthesis of Books and Illustrations Past and Present* (*Guijintushu jicheng*), 1728, has a few sections from Gong’s *Healing Myriad Illnesses* on topics such as ear problems and paediatrics, as well as poems on doctors and patients.^93^ The late-eighteenth-century imperially-commissioned *Emperor’s Four Treasuries* (*Siku quanshu*) also listed *Healing Myriad Illnesses* and *Effective Potions of Immortality* as part of Huang Yuji’s catalogue within the compilation.^94^ The limitations to Gong’s place in official compilations becomes much more clear upon comparison to his contemporary Xue Ji, who was featured much more prominently in official works.^95^

While Gong was less visible in Qing imperially commissioned texts, the state was not monolithic, and Gong’s texts were found in other areas of state medicine. At the Imperial Pharmacy, in addition to *Healing Myriad Illnesses*, there were copies of three more of his texts: *Effective Potions of Immortality, Ancient and Modern Mirror of Medicine*, and *Benefiting Society through the Enjoyment of Longevity.*^96^ Gong’s works at the Imperial Pharmacy were together with other popular medical works, classical Chinese medical texts, a Western medical treatise, and a text from Korea, Hŏ Chun’s (1539–1615) major compendium entitled *Precious Mirror of Eastern Medicine* (*Tongŭi pogam*, 1613).^97^

Moreover, Gong’s texts were also among those translated into Manchu during the Qing. Examples of Manchu medical manuscripts and blockprints of Gong’s works include the Manchu translation of *Benefiting Society through the Enjoyment of Longevity* from the Kangxi Emperor’s reign (1661–1722). The Manchu language text was entitled Ma. *Šeo ši boo iuwan*, transcription of the Chinese title *Shoushi baoyuan*.^98^ The extant Manchu medical texts weigh more heavily towards practical medical knowledge and less on medical theory. These are works on topics such as pulses, pharmacology, smallpox, acupuncture, gynaecology, hygiene, nutrition, forensic medicine, equine care, western drugs, as well as verses on medicinals etc. rather than texts which expound on medical theory.^99^ The existence of Gong’s work in Manchu translation, and also as part of the Chinese texts at the Qing Imperial Pharmacy demonstrate continuities between medicine in society and state medicine across dynastic lines.

In addition to Manchu manuscripts, texts housed at the Imperial Pharmacy, and works in state and society that referred to him, Gong’s visibility in the Qing dynasty continued through the printing of his texts, and within the materiality of drugs in the commercial pharmacy Tongren Tang.^100^ The Tongren Tang pharmacy was one of the commercial pharmacies in Beijing. Moreover, it also supplied drugs to the Imperial Pharmacy. The names of some medicines in their catalogue refer to Gong or the title of one of his works. For example, the section of the catalogue on medicines for children’s diseases has the compound medicine ‘*dan* from *Healing Myriad Illnesses*’ (*Wanbing huichun dan*).^101^ This drug clearly cites Gong’s famous text *Healing Myriad Illnesses*. Moreover a drug in the Stomach–Spleen section of the pharmacy catalogue was named ‘the Forest of Clouds’ pill for moisturizing the body’ (*Yunlin runshen wan*), referring to Gong’s literary name.

Although much of the analysis here has focused on the reception of Gong’s work across time, Gong’s texts also travelled across long distances. In a highly commercialised society with improved transportation and trade networks, knowledge easily travelled beyond the local context. The reception of Gong’s texts was not limited to China. The extent of intellectual and material exchange went much further, encompassing the region of East Asia.^102^ Yuming He has stated that producers and readers of texts during the Ming were aware of the regional breadth of print culture across East Asia.^103^ Therefore, while Gong’s work occupied a less-than prominent place within imperially commissioned texts, his texts also circulated in Japan and Korea.^104^ Gong’s *Healing Myriad Illnesses*, for example, had three Ming, 17 Qing, and 18 Japanese editions between 1611 and 1714.^105^ Research libraries around the world house a number of such Japanese editions of Gong’s texts, these include a 1636 edition of *Complete Book of Benefiting Society*, a 1645 Kyoto edition of his earlier work *Benefiting Society through the Enjoyment of Longevity*, a 1650 edition of *Effective Potions of Immortality*, as well as editions of *Healing Myriad Illnesses* from 1647 and 1660. Moreover, the catalogue by Ding Ren (1866–1920) lists a Ming edition of *Healing Myriad Illnesses*, as well as editions of *Benefiting Society through the Enjoyment of Longevity* and *Complete Book of Benefiting Society* that had both been printed in Japan.^106^

Gong’s work was not just known in Japan, but also in Korea. Soyoung Suh’s work on Hŏ Chun’s (1539–1615) major compendium *Precious Mirror of Eastern Medicine* (1613) references the reception of Gong Tingxian’s father Gong Xin’s *Ancient and Modern Mirror of Medicine* in Korea.^107^ The *Chosŏn Veritable Records* from the reign of Yeongjo (r. 1724–1776) note publication of an expanded edition of *Healing Myriad Illnesses* together with the very famous *Precious Mirror of Eastern Medicine* thereby attesting to the fame of Gong’s work in Korea.^108^ Furthermore, in an annotated bibliography from the Qing dynasty, the notes on the Korean edition of the *Precious Mirror of Eastern Medicine* stated that in Korea, the *Ancient and Modern Mirror of Medicine* and *Healing Myriad Illnesses* were attributed to Gong Tingxian’s father Gong Xin. Therefore, Gong’s work was received in Korea, even if it was not always with the correct attribution.^109^

Gong’s texts were not confined to East Asia either during this time. His *Healing Myriad Illnesses* was listed in the ‘Sinological Book Catalogue of the Brandenburg Electoral Library’ from 1685, where the Chinese title of the text ‘*Wanbing huichun*’ was recorded as ‘*Hoei-wam-bim-chun*’ with a note that it was a medical text. The same text continued to be listed in the 1822 catalogue of the Berlin Library by Klaproth.^110^ Gong’s *Healing Myriad Illnesses* in the Brandenburg Electoral Library was among other texts on matters of religion, mathematics, astronomy, medicine and Chinese dictionaries. The range of texts on religion and science are emblematic of the interests of early missionaries in China. Missionaries shared their scientific knowledge with the Chinese with an aim to convert them. At the same time, they also had specific interest in learning about Chinese medicine. The curiosity for Chinese understandings of the body can be seen through the 1682 publication of formulas for Chinese pulse medicine and a list of *materia medica* attributed to Dutch East India Company physician Andreas Cleyer. It was likely written by Jesuit Michael Boym with the list appended by Jesuit Philippe Couplet. Those involved in the publication process were most probably not limited to these figures alone. Boym’s contributions can therefore be understood within a broader range of work that Jesuits had been undertaking to learn and translate Chinese knowledge, including medicine.^111^ While Boym’s work reached Europe through Latin translation, Gong’s work was collected in Chinese.

##  Conclusion

In many ways, Gong’s attitude and actions reflected those attributed to men of his time. He resonated with ideals of the Confucian scholarly tradition as well as those in the highly commercialising society, and utilised these understandings to publicise his popular medical works. While he was not necessarily remembered for putting forth new medical ideas, he was skilled in synthesising a broad range of extant medical knowledge and presenting them in more easily accessible formats. Gong’s example shows the continuity of state–society relations as seen from the lens of a broad spatio-temporal frame. Temporally these extend from Ming to Qing, and geographically from China to Japan and Korea, as well as Europe.

Gong’s place in society constituted just one element of his medical identity. The reception of his work suggests that his state-connections were also an important part of his broader social medical personality. Yet it is also important to consider other factors that contributed to the popularity of his works across time and space. We have seen that while Gong occupied quite a central place in state medicine, this did not mean that he featured prominently in all official compilations. Other factors for the wider dissemination of his work include the breadth of medical issues covered in his texts, and his inclusion of treatments that were considered to be less prestigious but useful in practical terms. Presenting complex medical information in the form of easily memorisable songs or poems also facilitated the popularity of his texts. Extant Manchu language translations of medical texts are those of a practical nature, and the Manchu translations of Gong’s work are a case in point.

While this study focuses on a specific medical figure, it also has a number of broader implications. Gong’s example provides an opportunity to re-evaluate the relative emphasis accorded to state and society within accounts of medical popularisation in early modern China. An important prerequisite for recognising societal–state connections necessitates moving beyond assumptions of a unitary state that only exerts power top-down. Gong’s example shows the role that actors in society could play in utilising their state connections to further their wider societal aims.

Gong’s texts not only continued to be printed in China but were also well-received in East Asia and catalogued in Europe during the early modern period. The global nature of medicine in modern times has been clearly set out in the field.^112^ There is increasingly more work being done on medicine in a globalised early modern world.^113^ Gong’s medical popularisation, and his ensuing legacy with the cataloguing of his text in late seventeenth-century Europe provides further evidence for such geographical and cultural connectivities.^114^

This study revises the long held understanding that popularisation of medicine in early modern China took place within the context of the retreat of the state, and suggests that it was in fact a much more complicated and multifaceted process. Gong’s career, medical writings and legacy demonstrate that medical popularisation was not entirely in the private sphere and independent from a state retreating from medical governance. Rather in Gong’s case, his state connections furthered his medical popularising and his popularised medical works were conversely represented and found a place within textual collections in state medicine, as we can see through the Qing Imperial Pharmacy. Gong’s texts were not bound to state medicine either but were also found among the names of drugs within a commercial pharmacy which also happened to serve the Qing Imperial Pharmacy.

This study therefore underscores the methodological importance of using a broad temporal and spatial lens to reveal state–society continuities in medicine. Taking such an approach while also recognising the pluralities of state medicine is revealing of the way in which what at first glance seems to be an inherently local story situated in society at a particular juncture in time, also has continuities and ties to state medicine. And, perhaps even more unexpectedly, this approach shows how what is at heart a very local history also constitutes a thread in the wider entangled web of the global history of medicine and publishing in the early modern world.

## Footnotes

1 The term popular medicine and the process of popularisation in medicine have been defined in a number of different ways. While some have regarded popular as the opposite of elite, scholars have pointed to the limitations of such an understanding. Mary Lindemann, *Medicine and Society in Early Modern Europe* (Cambridge: Cambridge University Press, 2010), 16; Literature includes definitions that vary in the extent to which state-sanctioned knowledge plays a role in the process of popularisation, Roy Porter, ed., *The Popularization of Medicine 1650-1850* (London: Routledge, 1992), especially Roy Porter, ‘Introduction’, in Porter, ed., *The Popularization of Medicine*, 1–16, 3–4; Andrew Wear, ‘The Popularization of Medicine in Early Modern England’, in Porter, ed., *The Popularization of Medicine*, 17–41, 17, 37. In this article the term ‘popular’ is used interchangeably with primers and introductory texts, as seen in Leung (2003). The use of popular here also includes no assumption of state-sanctioned knowledge. See Angela K. C. Leung, ‘Medical Instruction and Popularization in Ming-Qing China’, *Late Imperial China*, 2003, *24*, 130–52, 130.

2 Scholars have debated the exact range of Gong’s lifetime. These include 1522–1619 and 1538–1635. For discussion on his year of birth and death Li Shihua *et al*., ‘Gong Tingxian Yixue Xueshusixiang Yanjiu’, in Li Shihua *et al*., eds, *Gong Tingxian yixue quanshu* (Beijing: Zhongguo Zhongyiyao Chubanshe, 1999), 1405–31, 1405–7; Fabien Simonis, ‘Mad Acts, Mad Speech, and Mad People in Late Imperial Chinese Law and Medicine’(unpublished PhD thesis, Princeton University, 2010), 276 fn. 39. This article takes the dates of Gong’s main published texts, 1581–1616, as a reference for when he was active during his lifetime.

3 For decreased state oversight on medicine see Angela K.C. Leung, ‘Organized Medicine in Ming-Qing China: State and Private Medical Institutions in the Lower Yangzi Region’, *Late Imperial China*, 1987, *8*, 134–66, 135; Chu Pingyi, ‘Calendrical Learning and Medicine, 1600-1800’, in Willard J. Peterson ed., *Cambridge History of China*, Vol. 9, Part 2 (Cambridge: Cambridge University Press, 2016), 372–411, 399. For question of ‘professionalisation’ see Yüan-ling Chao, *Medicine and Society in Late Imperial China: a Study of Physicians in Suzhou, 1600-1850* (New York: Peter Lang, 2009), 8–14. For boasting of palace doctors from one’s local area, see Leung, ‘Organized Medicine’, 151–2. A clear distinction between the prestige accorded to local medical bureaus and those at the capital can further clarify the argument.

4 While evaluating the relative emphasis on state and society, it is also important to consider scholarship that cautions against making such neat distinctions between the two. In the case of China, see for example, Cynthia J. Brokaw, ‘On the History of the Book in China’, in C. J. Brokaw and K. Chow, eds, *Printing and Book Culture in Late Imperial China* (London: University of California Press, 2005), 3–54; Joseph McDermott, ‘The Ascendance of the Imprint in China’, in Brokaw and Chow, eds, *Printing and Book Culture,* 55–104, 82–83; Yuming He, *Home and the World: Editing the “Glorious Ming” in Woodblock-Printed Books of the Sixteenth and Seventeenth Centuries* (Cambridge, MA: Harvard University Asia Center, 2013), 35.

5 Pluralities of medicine in society have been outlined by Volker Scheid, *Chinese Medicine in Contemporary China: Plurality and Synthesis* (Durham, NC: Duke University Press, 2002). Also see Bridie Andrews, *The Making of Modern Chinese Medicine, 1850–1960* (Vancouver: UBC Press, 2014). For pluralities in Qing state medicine within the context of cultural exchange, realigning hierarchies, and medical reform, see Sare Aricanli, ‘Diversifying the Center: Authority and Representation within the Context of Multiplicity in Eighteenth Century Qing Imperial Medicine’ (unpublished PhD thesis, Princeton University, 2016).

6 Porter, ‘Introduction’, 2; Matthew Ramsey, ‘The Popularization of Medicine in France’, in Porter, ed., *The Popularization of Medicine*, 97–133, 109–110; Charles E. Rosenberg, ‘“The Fielding H. Garrison Lecture”: Medical Text and Social Context: Explaining William Buchan’s “Domestic Medicine”’, *Bulletin of the History of Medicine*, 1983, *57*, 22–42.

7 Porter, ‘Introduction’, 3.

8
*Ibid.*, 8.

9 Stephen Greenblatt, *Renaissance Self-Fashioning: from More to Shakespeare* (London: University of Chicago Press, 1980).

10 Judith T. Zeitlin, ‘The Literary Fashioning of Medical Authority: A Study of Sun Yikui’s Case Histories’ in C. Furth, J.T. Zeitlin, and P. Hsiung, eds, *Thinking with Cases: Specialist Knowledge in Chinese Cultural History* (Honolulu: University of Hawaii Press, 2007), 169–204, 169, 176.

11 For Gong as author of primers in Leung, ‘Medical Instruction’, 135, 141, 148–49; Angela K.C. Leung, *Leprosy in China*: *a History* (New York: Columbia University Press: 2009), 40, 44. For Gong’s position in state medicine, and the wide distribution of his texts in China and their publication in Japan see Leung, ‘Medical Instruction’, 136. For Gong within the context of a study on madness in China, see Simonis, ‘Mad Acts, Mad Speech’, 265, 276-77.

12 His hometown is located in today’s Jiangxi province.

13 For Confucian benevolence and filial piety within the context of medicine, see for example Joanna Grant, *A Chinese Physician: Wang Ji and the ‘Stone Mountain’ Medical Case Histories* (London: Routledge, 2003), 49; Also see Zeitlin, ‘Literary Fashioning’, 176–77.

14 Grant, *A Chinese Physician*, 49, 156.

15 Gong Tingxian, *Jishi quanshu*, in Li Shihua *et al*., eds, *Gong Tingxian yixue quanshu*, 845–1088, 848. This study includes discussion of print-culture, a field in which matters such as manuscripts, type/script, publishers are all play an important role. However, the primary focus of this study is to examine continuities of Gong’s reception that can be seen through a re-examination of the relative emphasis to state and society in his texts. Therefore, a contemporary edition of Gong’s complete works, which allows easier access, has been used to reference his works.For sources of medical knowledge also see Simonis, ‘Mad Acts, Mad Speech’, 276. For the importance of locality and the geographic imagination in Chinese medicine, see Marta E. Hanson, *Speaking of Epidemics in Chinese Medicine: Disease and the Geographic Imagination in Late Imperial China* (New York: Routledge, 2011). Gong did not employ an actor’s category to refer to social spheres of medical knowledge to which he had access, but gestured towards his ability to overcome barriers to access.

16 Leung, ‘Medical Instruction’, 136. Another example of influences within the world of popular medical texts is provided by Grant. She explains that Xue Ji, among others, had quite an influence on Wang Ji. Grant, *A Chinese Physician*, 43–44.

17 Gong, *Jishi quanshu*, 848. The Taiyiyuan is also translated as Imperial Academy of Medicine. The translation here is Imperial Medical Bureau, as ‘academy’ overemphasises the aspect of learning, over an office of doctors who provided medical treatment.

18 Simonis, ‘Mad Acts, Mad Speech’, 277; Leung, ‘Medical Instruction’, 135–36 fn. 25.

19 Leung, ‘Medical Instruction’, 147–48. For the effect of merchant networks on the transmission of medical ideas, see Grant, *A Chinese Physician*, 34–5, 44; Zeitlin, ‘Literary Fashioning’, 170.

20 For a discussion of cultural reproduction, see Benjamin A. Elman, ‘Political, Social, and Cultural Reproduction via Civil Service Examinations in Late Imperial China’, *The Journal of Asian Studies*, 1991, *50*, 7–28, 14–23.

21 Medicine attained an increasingly scholarly characteristic through medical classics and rituals since medieval times. See Robert P. Hymes, ‘Not Quite Gentlemen? Doctors in the Song and Yuan’, *Chinese Science*, 1987, *8*, 9-76, 64–65; Leung, ‘Medical Instruction’, 135; Leung, ‘Organized Medicine’, 138, 152; also see Chao, *Medicine and Society*, 25–78. For discussion of the divergence between scholarly and popular medicine see Angela K.C. Leung, ‘Medical Learning from the Song to the Ming’ in P. J. Smith and R. Von Glahn, eds, *The Song-Yuan-Ming Transition in Chinese History* (London: Harvard University Asia Center, 2003), 374–82; Chu, ‘Calendrical Learning and Medicine’, 401.

22 Craig Clunas, *Superfluous Things: Material Culture and Social Status in Early Modern China* (Honolulu: University of Hawai’i Press, 2004); Timothy Brook, *The Confusions of Pleasure: Commerce and Culture in Ming China* (London: University of California Press, 1999); Grant, *A Chinese Physician*, Ch. 1, see esp. 26. Publishing also played a role in blurring the boundaries between societal groups, resulting in, for example, literati-merchant-businessmen (*shi-shang*). Kai-wing Chow, *Publishing, Culture, and Power in Early Modern China* (Stanford: Stanford University Press, 2004), 1–2, 98.

23 In addition to medical texts, publications included encyclopaedias, fortune telling and divination manuals, literary collections, jottings and notes, guides for travel, manuals of etiquette, almanacs. Brokaw, ‘On the History of the Book’, 11, 23–25.

24 The actor’s category in question is ‘*ruyi*’. The second character ‘*yi*’ could be ‘doctor(s)’ or ‘medicine’ in English, and generally refers to doctors. The first character ‘*ru*’ indicates that the doctor is literate, elite, part of the bureaucracy, and steeped in the classical textual tradition. While earlier scholarship has also referred to the category *ruyi* as Confucian doctor/physician, there has also been acknowledgement of the challenges with doing so, as ‘*ru*’ has been used loosely to refer to scholarly nature, association with an elite lineage, education, and morality. See Hymes, ‘Not Quite Gentlemen?’, 10, 13 fn.10, 14. Chu, ‘Calendrical Learning and Medicine, 401. For designations such as ‘literate practitioners’ or ‘literate healers recognisable as practitioners of classical Chinese medicine’ to refer to scholarly doctors, see Christopher Cullen, ‘Patients and Healers in Late Imperial China: Evidence from the Jinpingmei’, *History of Science*, 1993, *31*, 99–150, 106, 108, 115. For the Confucian primer, see Leung, ‘Medical Instruction’, 145. For Confucian doctors, also see Grant, *A Chinese Physician*, 41; Lucille Chia, ‘Of Three Mountains Street: The Commercial Publishers of Ming Nanjing’, in Brokaw and Chow, eds, *Printing and Book Culture*, 107–151, 136; Leung, ‘Medical Instruction’, 147. This article uses literati or scholarly to refer to the social category of ‘*ru*’ doctors/medicine.

25 For those who turned to medicine after failing in the civil service examinations, see Grant, *A Chinese Physician*, 31 fn. 92–93, 39; Leung, ‘Medical Instruction’, 135; Leung, ‘Medical Learning’, 392.

26 Leung, ‘Organized Medicine’, 152; Hymes, ‘Not Quite Gentlemen’, 23, 27–30, 40–68.

27 Gong Tingxian, *Wanbing huichun*, in Li Shihua *et al*., eds, *Gong Tingxian yixue quanshu*, 211-466*,* 215.

28
*Ibid.*, 215.

29
*Ibid.*, 215.

30 Joseph P. McDermott, *A Social History of the Chinese Book: Books and Literati Culture in Late Imperial China* (Aberdeen: Hong Kong University Press, 2006), 112–14. Joseph McDermott cautions against assuming a very early ascendance of the imprint vis-à-vis the manuscript, and suggests taking a regional approach to date it at the sixteenth-century lower-Yangtze delta. McDermott, *A Social History of the Chinese Book*, 49; McDermott, ‘Ascendance of the Imprint’, 56–59. Also see Brokaw, ‘On the History of the Book’, 16. The shift may also have been genre-specific, and later for fields such as medicine and play scripts. McDermott, *A Social History of the Chinese Book*, 44. For continuity of manuscript culture in medicine, also see Grant, *A Chinese Physician*, 44.

31 To mediate such difficulties, groups of book-collectors with no kinship-ties shared material with one another in a society where ties of kinship and learning were highly regarded. McDermott, *A Social History of Chinese Book*, Chs 4–5.

32 Medical texts were for example the largest category of books published in Jianyang, at 14 per cent followed by encyclopaedias. Lucille Chia, *Printing for Profit: the Commercial Publishers of Jianyang, Fujian (11th-17th centuries)* (Cambridge, MA: Harvard University Asia Center for Harvard-Yenching Institute, 2002), 312–13 (Appendix C, Table C.2). Medical texts constituted 10 per cent of texts published in Nanjing, and 15.3 per cent of all texts published in Jianyang. Chia, ‘Of Three Mountains Street’,129–30 (Table 3.3), 135. Not everyone published for profit, however. Some just aimed to accumulate moral capital. See Ellen Widmer, ‘The Huanduzhai of Hangzhou and Suzhou: A Study in Seventeenth-century Publishing’, *Harvard Journal of Asiatic Studies*, 1996, *56,* 77–122, 111–12. Publishers were also of varied backgrounds. Grant, *A Chinese Physician*, 36 fn. 120; McDermott, *A Social History of the Chinese Book*, 69.

33 Grant, *A Chinese Physician*, 37. Leung, ‘Medical Instruction’, 144.

34 Gong Tingxian, *Zhongxing xianfang*, in Li Shihua *et al*., eds, *Gong Tingxian yixue quanshu* , 1–64, 3; also see Rosenberg, ‘The Fielding H. Garrison Lecture’, 25.

35 Gong, *Wanbing huichun*, 215.

36 Leung, ‘Medical Instruction’, 133–46.

37
*Ibid.*,132–33.

38 Gong, *Wanbing huichun*, 381–431.

39 Gong Tingxian, *Lufu jinfang*, in Li Shihua *et al*., eds, *Gong Tingxian yixue quanshu* , 65–140, 138.

40 The term vernacular is also a challenge to define, especially when considered across cultural contexts. In Western languages, vernacular generally refers to the distinction between local languages and Latin. However, in the Chinese case, there are a number of linguistic registers (classical or literary, spoken) of Chinese with levels of literacy in between. In China, the unity of language is not compromised with publication in a less formal language. There is also the genre-specific element, which is that medical writings were often in the literary language of classical Chinese, see Zeitlin, ‘Literary Fashioning’, 171. On language and the challenges of designations of popular and vernacular see Brokaw, ‘On the History of the Book’, 11–13. There has also been discussion of technical or scientific works in local languages. Ramsey, ‘The Popularization of Medicine in France’, 99. For medieval times, see William Crossgrove, ‘The Vernacularization of Science, Medicine, and Technology in Late Medieval Europe: Broadening our Perspectives’, *Early Science and Medicine*, 2000, *5,* 47–63.

41 Chen Xiuyuan, *Yixue sanzijing* (Beijing: Renmin Weisheng Chubanshe, 1956), 14; Simonis, ‘Mad Acts, Mad Speech’, 277.

42 In the section entitled ‘on not reading ancient books’ Wu Tang stated that people turned to Gong’s work rather than reading the classics like the *Yellow Emperor’s Inner Canon* or the works of famous physicians such as Zhang Zhongjing. Wu Tang, *Zengding yiyi bingshu*, in H. Shen and X. Liang, eds, *Zhongguo lidai mingyi yihua daguan* (Taiyuan: Shanxi Kexue Chubanshe, 1996), 505–553, 519. Also see, Simonis ‘Mad Acts, Mad Speech’ 277. For the scholarly tradition of medicine with classical medical texts, see Leung, ‘Medical Learning’, 375–82. Medical classics should be differentiated from the classical texts sometimes referred to as the Confucian classics.

43 In the field of literature, Robert E. Hegel has examined identity as situated in the mind or body, Robert E. Hegel, *The Novel in Seventeenth-century China* (New York: Columbia University Press, 1981), 144, 147, 152, 155–66, 170–71. Gong wrote about medical treatment of the body while also claiming to have resonances with the scholarly tradition. Also see He, *Home and the World*, 73; Chow, *Publishing, Culture, and Power*, 100.

44 Gong, *Zhongxing xianfang*, 3. Also see *Jishi quanshu*, 848.

45 Gong, *Zhongxing xianfang*, 3; Gong, *Wanbing huichun*, 215.

46 Gong, *Wanbing huichun*, 215.

47 He travelled and worked in the vicinity of Beijing, as well as Hebei, Shanxi, Shaanxi, Shandong. Gong, *Jishi quanshu*, 848; For Suzhou and Nanjing, see Simonis, ‘Mad Acts, Mad Speech’, 276.

48 Gong was also recognised in a late-nineteenth century (1870) local official history (local gazetteer) of his hometown. Chen Fang *et al.*, eds, *Jinxi xianzhi* (Taibei: Zhongguo Fangzhi Congshu, Jianxi sheng, 1989), 1763, 1768.

49 Gong, *Zhongxing xianfang*, 3. Gong Tingxian, *Shoushi baoyuan*, in Li Shihua *et al*., eds, *Gong Tingxian yixue quanshu* , 467–844, 470.

50 Chow, *Publishing, Culture, and Power*, 113–14, 133.

51 Grant, *A Chinese Physician*, 33; Brokaw, ‘On the History of the Book’, 18; McDermott, ‘Ascendance of the Imprint’, 83.

52 Gong, *Lufu jinfang*, 67.

53 Gong Tingxian, *Yunlin shengou*, in Li Shihua *et al.*, eds, *Gong Tingxian yixue quanshu* , 141–210, 143; Also see Gong, *Wanbing huichun*, 213. For Maokun see Zhang Tingyu, *Mingshi* (Beijing: Zhonghua Shuju, 1974), *juan* 287, 7374–75; Huizhi Zhang, *Zhongguo lidai renming dacidian* (Shanghai: Shanghai Guji Chubanshe, 1999), vol. 2, 1436.

54 Gong, *Yunlin shengou*, 143.

55 Leung, ‘Medical Learning’, 377–82.

56 Gong, *Yunlin shengou*, 143. See section below on ‘Zuo Si.’

57 For secret knowledge in medicine, see for example, Zeitlin, ‘Literary Fashioning’, 177.

58 For a brief introduction to preparation for examinations, see Elman, ‘Political, Social, and Cultural Reproduction’, 16–17.

59 Gong, *Wanbing huichun*, 215.

60 Gong, *Zhongxing xianfang*, 3.

61 Gong, *Jishi quanshu*, 470.

62 See biography of Zuo Si in *Jinshu* (Beijing: Zhonghua Shuju, 1974), *juan* 92, 2375–76.

63 While the scholarly medical tradition was often projected as one which was literary and distinct from Daoist practices and understandings, these worlds were not always neatly delineated. For a scholarly doctor who could also provide a commentary on a classical medical text through understandings of Daoist inner alchemy, see Leslie de Vries, ‘The Authentic Person as Ideal for the Late Ming Dynasty Physician’, *Synthesis Philosophica*, 2014, *29*, 63–82. For medicine and Daoism, also see Zeitlin, ‘Literary Fashioning’,177; Leung, ‘Medical Learning’, 391.

64 The proximity of Gong’s hometown Jinxi to Mount Longhu became clear upon a visit to Gong’s village. Mount Longhu was the headquarters of the Celestial Masters which also had imperial connections since the Ming, see Livia Kohn, ed, *Daoism Handbook* (Leiden: Brill, 2000), 641–42.

65 Gong, *Zhongxing xianfang*, 3.

66
*Ibid.*, 3; Robert Ford Campany, *To Live as Long as Heaven and Earth: A Translation and Study of Ge Hong’s Traditions of Divine Transcendents* (Ewing, NJ: University of California Press, 2002), 141–46. Xiao Tianshi, *Lidai zhenxian* *shizhuan*, Historical biography 2 (Taibei: Ziyou Chubanshe, 1960), 26–27.

67 For a drug name with Yunlin, see *Tongren Tang yaomu* (Tongzhi reign text. Preface dating 14 April 1869 (TZ8/3/3) therefore 1869–1875), 4a; Gong, Zhongxing xianfang, 5; Chen Keji and Li Chunsheng, eds, *Zhongguo gongting yixue* (Beijing: Zhongguo Qingnian Chubanshe, 2003), 407.

68 Gong Tingxian, *Yunlin shengou*, 24cm, dated 1591 to 1644 (Maokun’s preface dated 1591), 19th century print. The title page reads *Imperial Medical Bureau Divine Archer’s Pills of Immortality* (*Taiyiyuan shengou jindan*).

69 Gong, *Wanbing huichun*, 215.

70 Gong, *Zhongxing xianfang*, 3; Zeitlin, ‘Literary Fashioning’, 177.

71 Li Shihua, *et al*., ‘Gong Tingxian Yixue Xueshusixiang’,1416, 1425–28. For ‘ghosts’ see Chen Xiufen, ‘Dang Bingren Jian Dao Gui: Shilun Mingqing Yizhe dui yu ‘Xiechong’ de Taidu’, *Guoli zhengzhi daxue lishi xuebao*, 2008, *30*, 43–86.

72 Gong, *Zhongxing xianfang*, 3.

73
*Ibid.*, 3.

74 Gong, *Wanbing huichun*, 215.

75 When an official at the Imperial Medical Bureau had a son, grandson, or nephew by the male line, and the institution had positions that were vacant, the Ministry of Rites would accept them for examination. See the newly revised Ming *Collected Statutes* from the Wanli era (1585), *Da Ming huidian* (Taibei: Taiwan Shangwu Yinshuguan, 1968), 4417–18.

76 Chen Keji and Li Chunsheng, eds, *Zhongguo gongting yixue*, 406.

77
*Ibid*., 407.

78 Gong, *Jishi quanshu*, 848; Gong, *Lufu jinfang*, 67.

79 For more detail on ranks and their distribution across departments, see *Da Ming huidian*, 4414.

80 Gong, *Shoushi baoyuan*, 470. Also see Gong, *Jishi quanshu*, 848.

81 Gong, *Shoushi baoyuan*, 470.

82
*Ibid.*, 470.

83 For general regulations (*fanli*), see Chia, ‘Of Three Mountains Street’, 126; Widmer, ‘The Huanduzhai of Hangzhou and Suzhou’, 102–3.

84 Gong, *Shoushi baoyuan*, 471. Emphasis added.

85 Moxibustion is a therapeutic method used by holding a smoking stick of moxa (*artemisia vulgaris*) on acupuncture points or meridians, and burning it directly (or with some intermediary substance) above that point on the skin. Zhongyi yanjiuyuan and Guangdong zhongyi xueyuan, eds, *Zhongyi mingci shuyu cidian* (Hong Kong: Shangwu Yinshuguan, 1976), 304. External, manual therapies, including acupuncture and moxibustion, were regarded as being unorthodox, lower in status, marginal, and technical in nature, in relation to literati medicine that favoured prescriptions of drugs. Practitioners of acupuncture and moxibustion also included many female healers. Leung, ‘Medical Instruction’, 134, 138; Leung, ‘Medical Learning, 383–85, 396 fn. 90; Zeitlin, ‘Literary Fashioning’, 194 fn. 63, 210 fn. 63; Chao, *Medicine and Society*, 35 fn. 31.

86 Gong, *Wanbing huichun*, 215.

87 Gong Tingxian, *Xiao’er tuina fangmai huoying mizhi quanshu*, in Li Shihua *et al*., eds, *Gong Tingxian yixue quanshu*, 1089–134, 1091.

88 See for example, medical figures Zhang Jiebin, Li Shizhen, Xue Ji, Li Zhongzi, Zhang Tingyu, in *Mingshi*, *juan* 98, 2447–48. For Li Shizhen’s biography, see Zhang Tingyu, *Mingshi*, *juan* 299, 7653.

89 Wan Sitong, *Mingshi*, 416 *juan*, in *Xuxiu siku quanshu*, *shi bu*, *bieshi lei*, vol. 326 (Shanghai: Shanghai Guji Chubanshe, 2008), 432.

90 Huang Yuji, *Qianqingtang shumu* (Beijing: Wenwu Chubanshe, 1992), 12a.

91 For writing of the official Ming history, and differences between Draft version of Ming dynastic history and later versions, see Achim Mittag, ‘Chinese Official Historical Writing under the Ming and Qing’ in José Rabasa *et al.*, eds, *The Oxford History of Historical Writing: Volume 3: 1400-1800* (Oxford: Oxford University Press, 2012), 24–42, 30–32.

92 Zhou Zhongfu, *Zhengtang dushuji* (Beijing: Zhonghua Shuju, 1993), *juan* 43, 785.

93 There are a number of references to Gong’s *Wanbing huichun*, see *Gujintushu jicheng* (Shanghai: Shanghai Tushujichengju, [1885–1888])*, minglun renshi juan 31 chusheng 13, bowu yishu juan 154 yibu* 134, *bowu yishu juan 184 yibu 164*, *bowu yishu juan 422 yibu 420*, *bowu yishu juan 428 yibu 408.*

94 For the *Emperor’s Four Treasuries*, see R. Kent Guy, *The Emperor’s Four Treasuries: Scholars and the State in the Late Ch’ien-lung Era* (Cambridge: Harvard University Press, 1987). For the reference to Gong, see *Siku quanshu* (Taipei: Taiwan Shangwu Yinshuguan, 2013), vol. 676, *juan* 14, 18a.

95 Xue Ji, for example, figures very prominently in more official sources. Zhang Tingyu, *Mingshi*, *juan* 98, 2447. Xue Ji’s case records, *Xueshi yi’an* is within the *Emperor’s Four Treasuries*, *Siku quanshu*, vols. 763–64. There are dozens of references to his case records written as *Xueji yi’an*, see for example *Gujintushu jicheng*, *bowu yishu juan* 114 *yibu* 94, *juan* 143 *yibu* 123, *juan* 150 *yibu* 130, *juan* 153 *yibu* 133, *juan* 157 *yibu* 137, *juan* 170 *yibu* 150, *juan* 172 *yibu* 152, *juan* 174 *yibu* 154. For more on Xue Ji, also see Yi-Li Wu, ‘Body, Gender, and Disease: The Female Breast in Late Imperial Chinese Medicine’, *Late Imperial China*, 2011, *32,* 83–128.

96
*Yuyaofang yishu zongdang*, QL32. Also see Chen Keji *et al.*, eds, *Qingdai gongting yihua, Xiandai zhuming laozhongyi mingzhu chongkan zongshu* no. 7 (Beijing: Renmin Weisheng Chubanshe, 2012), 23–27.

97 Soyoung Suh, *Naming the Local: Medicine, Language, and Identity in Korea since the Fifteenth Century* (Cambridge, MA: Harvard University Asia Center, 2017).

98 A prescription, such as the ‘decoction for rectifying *qi*’ (*zhengqi tang*), lists ingredients as transcriptions of Chinese characters written in Manchu, such as Ma. *cing pi* (pronounced ching-pi) for Ch. *qingpi*, Ma. *fu ling* for Ch. *fuling*, etc. Therefore, the Manchu texts in the imperial medical world created a new vocabulary of medicinals through transcriptions into Manchu. Ma. *Šeo ši boo iuwan* (translation of Ch. *Shoushi baoyuan* (*Benefiting Society through the Enjoyment of Longevity*), [Qing], MS. For corresponding section see Gong, *Shoushi baoyuan*, 561.

99 For a detailed and annotated list of Manchu medical manuscripts, see Marta E. Hanson, ‘On Manchu Medical Manuscripts and Blockprints: An Essay and Bibliographic Survey’, *Saksaha: a Review of Manchu Studies*, 2003, *8,* 1–32, 13–14. Chen, *Gongting yihua*, 25.

100 For the history of the Tongren Tang see Beijing Tongren Tang Jituan Gongsi yu Beijing Tongrentangshi Bianweihui, *Beijing Tongren Tang shi* (Beijing: Renmin Ribao Chubanshe, 1993); Sherman Cochran, *Chinese Medicine Men: Consumer Culture in China and Southeast Asia* (Cambridge, MA: Harvard University Press, 2006), Ch. 2.

101
*Dan* is a kind of medicine that can be taken orally or externally, where external form includes mineral drugs with mercury and sulphur in form of powder. Yuan Yixiang *et al.*, eds, *Chinese-English Dictionary of Traditional Chinese Medicine* (Beijing: Renmin Weisheng Chubanshe, 1997), 197. These are mineral drugs that can be in powder, pellet, or cake form. Also referred to as ‘magic potion of immortals’ (*lingyao*), these are made from metallic and mineral substances that are sublimated and extracted, reflecting the connections between the medicine and Daoism. Zhongyi yanjiuyuan and Guangdong zhongyi xueyuan, eds, *Zhongyi mingci shuyu cidian*, 276, 263. *Tongren Tang yaomu* (Guangxu reign, 1889), table of contents, pediatrics section; *Tongren Tang yaomu* (Tongzhi reign), 4a.

102 Scholars have demonstrated the value of examining historical connections across East Asia. With respect to intellectual history, see Benjamin A. Elman, John B. Duncan, and Herman Ooms, eds, *Rethinking Confucianism: Past and Present in China, Japan, Korea, and Vietnam* (Los Angeles: UCLA Asian Pacific monograph Series, 2002). For history of medicine, see Benjamin A. Elman, ‘Sinophiles and Sinophobes in Tokugawa Japan: Politics, Classicism, and Medicine During the Eighteenth Century’, *East Asian Science, Technology, and Society: an International Journal*, 2008, 2, 93–121. Also see special journal volume Marta E. Hanson, eds, ‘New Approaches to the History and Anthropology of Korean Medicine’, *Asian Medicine*, 2016, 11. For an interactive history of early modern China through political, economic, and cultural exchanges across Northeast Asia, see Evelyn S. Rawski, *Early Modern China and Northeast Asia: Cross-border Perspectives* (Cambridge: Cambridge University Press, 2015).

103 He, *Home and the World*, 42. Joanna Grant also lists a number of texts from the prosperous mercantile region of Huizhou that not only circulated in China, but that were also sent to Korea and Japan. These include works by Wang Ji and Wang Ang. Grant, *A Chinese Physician*, 35, 45.

104 Also see, He, *Home and the World*, 42.

105 Leung, ‘Medical Instruction’, 135–36 fn. 25. Simonis, ‘Mad Acts, Mad Speech’, 277.

106 Ding Ren, *Baqianjuanlou shumu* (Qiantang, 1923), *juan* 10, 29a.

107 Suh, *Naming the Local*, 57–58.

108
*Chosŏn wangjo sillok* (Sŏul: Kuksa P’yŏnch’an Wiwŏnhoe, [1955- 1963]), *juan* 80, 12b. For the *Precious Mirror of Eastern Medicine*, see Suh, *Naming the Local*.

109 Zhou Zhongfu, *Zhengtang dushuji* (Beijing: Zhonghua Shuju, 1993), *juan* 43, 794.

110 See Andreas Müller, *Speciminum Sinicorum Andrae Mülleri Greiffenhagii Decimae de Decimis: una cum Mantissis* [1685]. https://archive.thulb.uni-jena.de/ufb/rsc/viewer/ufb_derivate_00001790/Math-2-48-1_028.tif [accessed 4 August 2019]. Also see catalogue from 1822 with Chinese books and manuscripts at the Berlin. Julius Klaproth, *Verzeichniss der chinesischen und mandshuischen Bücher und Handschriften der Königlichen Bibliothek zu Berlin*https://digital.staatsbibliothek-berlin.de/werkansicht/?PPN=PPN717737985&PHYSID=PHYS_0190 [accessed 31 October 2019]

111 Marta Hanson and Gianna Pomata, ‘Medicinal Formulas and Experiential Knowledge in the Seventeenth-Century Epistemic Exchange between China and Europe’, *Isis*, 2017, 108, 1–25; Beatriz Puente Ballesteros, ‘Jesuit Medicine at the Kangxi Court (1662-1722): Imperial Networks and Patronage’, *East Asian Science, Technology, and Medicine*, 2011, 34, 86–162; For translation of anatomy from French to Manchu by Jesuits at the Qing court, see Aricanli, ‘Diversifying the Center’, Ch. 5.

112 Mark Harrison, ‘A Global Perspective: Reframing the History of Health, Medicine, and Disease’, *Bulletin of the History of Medicine*, 2015, *89,* 639–89.

113 Harold J. Cook and Timothy D. Walker, eds, ‘Mobilizing Medicine: Trade and Healing in the Early Modern Atlantic World’, special issue, *Social History of Medicine*, 2013, 26, 337–431.

114 Also see He, *Home and the World*, 14–15.

